# Monitoring Energy Balance in Breast Cancer Survivors Using a Mobile App: Reliability Study

**DOI:** 10.2196/mhealth.9669

**Published:** 2018-03-27

**Authors:** Mario Lozano-Lozano, Noelia Galiano-Castillo, Lydia Martín-Martín, Nicolás Pace-Bedetti, Carolina Fernández-Lao, Manuel Arroyo-Morales, Irene Cantarero-Villanueva

**Affiliations:** ^1^ Department of Physical Therapy University of Granada Granada Spain; ^2^ Centro de Investigación Deporte y Salud University of Granada Granada Spain; ^3^ Instituto de Investigación Biosanitaria ibs-Granada Complejo Hospitalario Universitario de Granada University of Granada Granada Spain

**Keywords:** telemedicine, breast neoplasms, survivors, life style, exercise, diet, mhealth

## Abstract

**Background:**

The majority of breast cancer survivors do not meet recommendations in terms of diet and physical activity. To address this problem, we developed a mobile health (mHealth) app for assessing and monitoring healthy lifestyles in breast cancer survivors, called the Energy Balance on Cancer (BENECA) mHealth system. The BENECA mHealth system is a novel and interactive mHealth app, which allows breast cancer survivors to engage themselves in their energy balance monitoring. BENECA was designed to facilitate adherence to healthy lifestyles in an easy and intuitive way.

**Objective:**

The objective of the study was to assess the concurrent validity and test-retest reliability between the BENECA mHealth system and the gold standard assessment methods for diet and physical activity.

**Methods:**

A reliability study was conducted with 20 breast cancer survivors. In the study, tri-axial accelerometers (ActiGraphGT3X+) were used as gold standard for 8 consecutive days, in addition to 2, 24-hour dietary recalls, 4 dietary records, and sociodemographic questionnaires. Two-way random effect intraclass correlation coefficients, a linear regression-analysis, and a Passing-Bablok regression were calculated.

**Results:**

The reliability estimates were very high for all variables (alpha≥.90). The lowest reliability was found in fruit and vegetable intakes (alpha=.94). The reliability between the accelerometer and the dietary assessment instruments against the BENECA system was very high (intraclass correlation coefficient=.90). We found a mean match rate of 93.51% between instruments and a mean phantom rate of 3.35%. The Passing-Bablok regression analysis did not show considerable bias in fat percentage, portions of fruits and vegetables, or minutes of moderate to vigorous physical activity.

**Conclusions:**

The BENECA mHealth app could be a new tool to measure energy balance in breast cancer survivors in a reliable and simple way. Our results support the use of this technology to not only to encourage changes in breast cancer survivors' lifestyles, but also to remotely monitor energy balance.

**Trial Registration:**

ClinicalTrials.gov NCT02817724; https://clinicaltrials.gov/ct2/show/NCT02817724 (Archived by WebCite at http://www.webcitation.org/6xVY1buCc)

## Introduction

Although the relationship between diet, physical activity, and health is widely known, excess energy intakes (diet) and sedentary lifestyles are common negative habits in cancer survivors [1]. This energy imbalance may not only be highly associated with the increased risk of incidence of some of the most frequent types of cancer, but they may also be determinants in the appearance of new cancers, the increase of relapses, and even mortality due to cancer [2,3].

International guidelines for cancer survivors include maintaining a healthy weight, limiting the consumption of high-calorie foods, and engaging in physical activity [4,5], together known as energy balance. Unfortunately, only 20% to 32% of cancer survivors adhere to these standards [6,7]. Thus, the development of feasible, reliable, and accurate diet and physical activity assessment methods, as well as the promotion of cost-effective personalized behaviors are necessary to improve adherence to healthy lifestyles.

Currently, the gold standard instruments for measuring physical activity levels and diet in different populations include accelerometry and direct observation, daily records, and 24-hour dietary recall, respectively [8,9]. Despite their widespread use, new evaluation strategies are necessary to ensure that they (1) are less time consuming for patients and researchers; and (2) do not require the presence of a specialist.

Information and communication technologies are emerging as new methods to accurately and remotely evaluate different pathological processes [10-13], including oncology [14]. Literature has reported the use of electronic health (eHealth) tools that collect data on or that promote healthy lifestyles using the internet and Web-based programs [15-20]. Even though some eHealth programs were used in studies with patients with cancer [21-24], none of them quantified energy balance.

Mobile health (mHealth) apps offer many advantages over eHealth systems, including (1) instantaneous and personalized feedback; (2) self-directing data collection; (3) user-friendly interfaces; (4) evaluator bias reductions; and (5) lower costs by reducing face-to-face procedures [25]. To date, several mHealth apps have been developed to promote healthy lifestyles in the general population [26-30], and for some pathologies, such as cardiac rehabilitation [31], weigh loss interventions for endometrial carcinoma [32], and exercise and nutrition counseling for breast cancer survivors [33]. However, no mHealth app has been developed specifically for breast cancer survivors that simultaneously records energy balance (intake and physical activity), and provides immediate energy balance feedback.

The Energy Balance on Cancer (BENECA) mobile app, developed to help breast cancer survivors overcome energy balance challenges, aims to motivate and sensitize breast cancer survivors to adhere to fully personalized physical exercise programs and nutritional plans in compliance with the international guidelines for cancer survivors. Here, we describe the development of the BENECA system, its test-retest reliability, and concurrent validity against the gold standard methods to assess diet and physical activity.

## Methods

### Overview

A descriptive reliability study was used to test inter- and intrarater responses for a novel mhealth assessment app for energy balance in breast cancer survivors. The app, BENECA mHealth system, was developed by the CUIDATE research group.

### Participants, Sample, and Procedures

Breast cancer survivors were enrolled from the Complejo Hospitalario Universitario in Granada, Spain, following their oncologist’s suggestion to join the test-retest reliability study between September 2016 and December 2016. Cancer survivors were eligible if (1) they had been diagnosed with breast cancer (estrogen-receptor-positive [ER+]); (2) had a body mass index (BMI) higher than 25 kg/m^2^; (3) were between 30 and 75 years old; (4) had basic abilities to use mobile apps; and (5) had completed their cancer treatment (adjuvant therapy) at least 6 months prior. The participants were excluded if they had chronic diseases or orthopedic issues that could interfere with their ability to walk. The project followed the Declaration of Helsinki guidelines and Law 14/2007 on biomedical research [34]. The study was approved by the local ethics committee of the Andalusian Health Service. All participants provided written informed consent.

A total of 20 patients was estimated to be necessary to achieve 90% power, to identify a correlation coefficient of 0.8 between the evaluation methods (gold standard versus the BENECA mHealth app), and to have an alpha error of 5%. Previous studies on the agreement between remote assessment methods had comparable sample sizes [12,14,35]. Taking into account potential study dropouts, 25 patients were invited to participate in this study. A pilot study was carried out with 10 healthy participants to develop, test, and improve the BENECA mHealth system. The data from the pilot study were not included in this study.

The participants attended the Sport and Health Center in Granada. A member of the research team downloaded the BENECA mHealth system app to the patient's mobile phone. The patients were asked to use the mHealth app at least once in the presence of a research team member to ensure the correct use of the system and ask questions if needed. Each participant was also equipped with a tri-axial accelerometer (ActiGraphGT3X+, Pensacola, FL, US). A specialized nutritionist with 3 years of experience with patients with cancer recorded the participant's sociodemographic data and their diet from the previous day using 24-hour dietary recalls. The participants also received 4 daily dietary record questionnaires, which they completed on 4 of the working days. When necessary, a member of the CUIDATE group telephoned participants if they were having difficulties with the BENECA mHealth system.

### Gold Standard Methods

#### Physical Activity

An accelerometer was used to assess the level of physical activity of the participants following a previously published protocol [36]. The patients received a daily questionnaire and were equipped with pre-programmed accelerometers (tri-axial accelerometer, ActiGraphGT3X+, Pensacola, FL, US). They were instructed to wear the accelerometer for 24 hours for 8 consecutive days. Only records obtained from 4 or more days of use (excluding the first day) and at least 10 hours of recording (1 minute intervals) per day were analyzed. The accelerometer data were blinded to the participants.

#### Dietary Habits

The gold standard method for measuring diet is direct observation. However, in this study, direct observation of the participants’ dietary habits was not feasible. Therefore, together with the diet information, 24-hour dietary recalls and dietary records were used as references [9]. With 4dietary records and 2, 24-hour dietary recalls, the intake of 6 days, with 5 eating occasions per day, could be collected.

##### Twenty Four-Hour Dietary Recalls

The 24-hour dietary recalls were obtained through interviews. The participants did not know in advance when they would be contacted. The specialized nutritionist asked, either in person or by phone [37], about their dietary intakes on the previous day. On the day of the evaluation, an interviewer (trained dietitian) systematically collected detailed information on the diet in the preceding 24 hours. The nutritional value (energy and macronutrients) was evaluated using the Alimentación y Salud software, version 2.0 (Instituto de Nutrición, Universidad de Granada, Spain).

##### Dietary Records

Due to their validity, dietary records are considered one of the best systems to evaluate dietary intake. These records are a kind of diary in which the patient must log all the food and beverages consumed during a full day [9]. Four dietary records were completed, coinciding with the accelerometer wearing time.

### Description of the BENECA Mobile Health System

**Figure 1:**
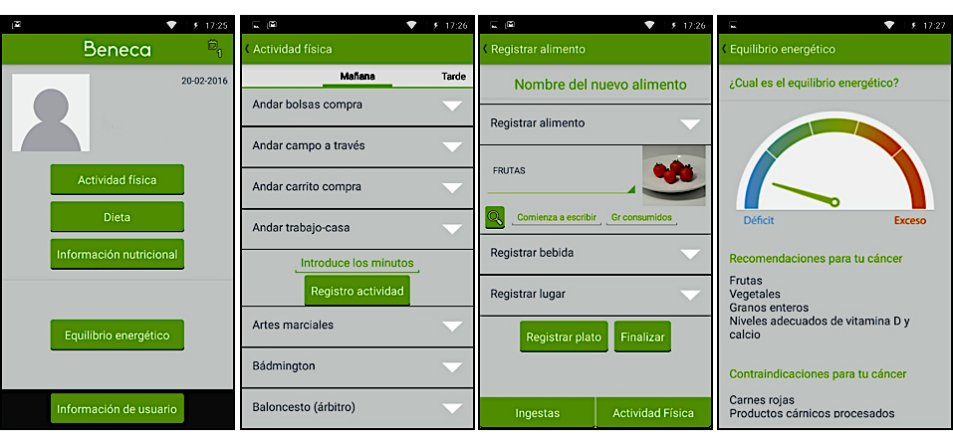
Screenshots of the Energy Balance on Cancer (BENECA) mobile health system.

The BENECA system was developed by the CUIDATE group, which consists of physiotherapists, occupational therapists, physical activity professionals, nutritionists, and a sports physician. BENECA is a native-Android mobile app (Figure 1), with a commercial server and centralized data storage. Its internal technological development has been described previously [38].

On first use, the users of the app record their personal and anthropometric data, such as weight, height, age, and type of cancer. They are then asked to record what they ate (every item) and what they did (in terms of physical activity) the day before. Regarding intake, BENECA uses a dietary record questionnaire, structured with 6 consumption times. On each day, for each period, users report all food and beverages taken. The app limits the food and drink options that can be selected, based on an internal, predefined list adjusted from the Spanish food database (Agencia Española de Seguridad Alimentaria y Nutrición/Base de Datos Española de Composición de Alimentos v1.0; 2010). The users are asked to record the most alike possibility offered if the food or drink is not on the predefined list.

The BENECA mHealth system was created from the validated Spanish version of the Minnesota Leisure-time Physical Activity Questionnaire [39]. The patients can record the activities completed during the day (intensity and duration), from 3 possible time periods (morning, afternoon, and evening). BENECA only records those activities that have a duration of at least 10 minutes. Internally, the app assigns a metabolic equivalent value (MET) to each activity based on the Compendium of Physical Activities [40].

Once the diet and physical activity are recorded, the users receive a daily straightforward notification about their energy balance, detecting if there has been an imbalance. Moreover, considering their individual profile and the information entered onto the BENECA mHealth app, the users can also obtain physical activity and dietary recommendations based on the guidelines of the World Cancer Research Fund International (WCRF), the strategies for physical activity and diet in patients with cancer from the American College of Sports Medicine [41], and the recommendations of the American Cancer Society [42]. A tutorial video of the BENECA mHealth app can be found in Multimedia Appendix 1.

### Statistical Analysis

For each outcome measure—minutes of moderate-to-vigorous physical activity, number of portions of fruits and vegetables, and percentage of fat—the agreement between gold standard assessment methods and the mHealth system was calculated. To evaluate a systematic change in the mean (bias) from test to retest, the mean difference with 95% CI was used. Moreover, we used 2-way random effect intraclass correlation coefficients (with their CIs) to the interrater reliably trials.

The agreement between diet (foods and drinks) recorded by BENECA and those reported in gold standard diet evaluation approaches were estimated based on the analysis reported previously by Hillier et al [10,11]. Match rates (food or drink items reported in gold standard methods that had also been recorded by the BENECA mHealth system), and phantom rates (items reported in gold standard methods that had not been recorded by the BENECA mHealth system), were calculated following the formulas described by Hillier et al [10].

Mean daily values of percentage of fat, portions of fruits and vegetables, and moderate-to-vigorous physical activity reported by BENECA were calculated for a concurrent validity analysis. The accuracy of the mHealth system was calculated using a linear regression analysis, and the correlation coefficient was determined. Finally, a Passing-Bablok regression was used to control bias [10]. IBM SPSS version 20 was used for all analyses (IBM Statistical Program for Social Sciences SPSS Statistic, Corp., Armonk, NY), and XLSTAT was used for Apple computers (2016 version, Addinsoft SARL).

## Results

### Test-Retest Reliability

The data obtained with each assessment method (gold standard versus BENECA mHealth system), and the mean differences are shown in Table 1. The mean difference of each outcome measure (gold standard versus BENECA mHealth system) and its alpha reliability estimate are also shown in Table 1. The reliability estimates in all analyses were high (alpha≥.90); portions of fruits and vegetables achieved the lowest reliability estimate with an alpha value of .94. The interrater intraclass correlation coefficients for each gold standard method and the BENECA mHealth system showed evidence of very good interrater reliability (intraclass correlation coefficient≥.90) (Table 1).

### Concurrent Validity

A total of 21 breast cancer survivor participants were recruited for this study. Of the participants, 1 (1/21, 5%) could not be included in the final sample because the Android version of her phone was not compatible with the BENECA system. Therefore, the final study sample consisted of 20 participants, with a mean age of 47.5 (SD 7.07) years.

The mean BMI of the sample was 26.51 (SD 3.06) kg/m^2^. Of the participants, 12 (12/20, 60%) had higher education, of which only 2 (2/20, 10%) had sick leave. The most commonly affected side was the right breast (11/20, 55%), and both breasts were affected in only 10% (2/20) of the survivors. Most of the participants were right-handed (18/20, 90%). Of the participants, 55% (11/20) had stage II breast cancer, and 20% (4/20) had stages I and IIIA.

**Table 1:** Cronbach alpha reliability estimates and interrater reliability between the gold standard measurement and the Energy Balance on Cancer (BENECA) mHealth system. ICC: intraclass correlation coefficient.

Variable		Mean difference between methods in units of measurement, 95% CI	Cronbach alpha reliability estimate interrater	Interrater reliability ICC	
				ρ^a^	95% CI
**Percentage of fat**						
	Total	0.15 (–1.44 to 1.74)	.956	.916	0.80 to 0.97
	Dietary record	1.32 (0.23 to 2.4)	.957	.918	0.81 to 0.97
	24-hour dietary recall	0.29 (–0.99 to 1.59)	.985	.971	0.93 to 0.99
**Portions of fruits and vegetables**						
	Total	0.01 (–0.22 to 0.23)	.982	.964	0.91 to 0.99
	Dietary record	–0.07 (–0.44 to –0.30)	.948	.901	0.77 to 0.96
	24-hour dietary recall	0.26 (–0.11 to 0.63)	.970	.941	0.85 to 0.97
Minutes of moderate-to-vigorous physical activity		8.89 (6.16 to 11.64)	.991	.982	0.95 to 0.99

^a^ICC (ρ) was calculated using a 2-way mixed effect model.

A unilateral mastectomy and a lumpectomy had been performed on 40% (8/20) and 50% (10/20) of the participants, respectively. Only 2 (10%, 2/20) participants underwent a bilateral mastectomy. In addition, 75% (15/20) received postsurgical adjuvant radio-chemotherapy, and 75% (15/20) were also receiving hormonal therapy (the estrogen receptor antagonist tamoxifen).

### Compliance With Methods

Paired data for the comparison between the BENECA mHealth system and the dietary records or accelerometer were collected for all participants. The compliance rates for all assessment methods were very high. All participants completed the BENECA system on the 6 requested days. In addition, 18 participants (90%, 18/20) completed the BENECA system on more days than requested. Similarly, compliance with the gold standard assessment methods was 100%. Breast cancer survivors completed the 4 dietary records and the 2, 24-hour dietary recalls; they also wore the accelerometer for the 8 requested days. Compliance with the accelerometer was very good; there were no incomplete sets of data, and the participants did not report any problems with the device (ie, allergic skin reactions).

The BENECA mHealth system showed excellent agreement with both dietary evaluation approaches (Table 2). The dietary records and 24-hour dietary recalls showed high match rates and low phantom rates. There were 30 intake times and 1630 diet items recorded; only 106 items were not recalled in the BENECA system (omitted or forgotten). “Vegetables” was the most frequently ignored item, followed by biscuits and crisps. Of the total, there were 21 (1.29%, 21/1630) occasions in which the food was not available on the BENECA system. In most of these cases the food items were replaced by an appropriate alternative from the BENECA food option list. However, some food items, such as “couscous,” were not replaced, and the choices were entered as “matches” for replaced items. Fifty nine “phantom” items were recorded in the BENECA system without being recorded in the gold standard dietary assessment methods, with biscuits and sweets being the most common “phantom” items.

No significant differences were found between the BENECA mHealth system and the gold standard assessment methods regarding percentage of fat compared to the 24-hour dietary recall (Table 3).

**Table 2:** Food item agreement between the Energy Balance on Cancer (BENECA) app and the gold standard dietary instruments.

Day		Match rate	Phantom rate
**Dietary record (%)**				
	1	94.41	5.63
	2	88.87	2.53
	3	89.04	2.02
	4	94.01	19.10
	Mean (SD)	91.58 (9.55)	7.32 (14.96)
**24-hour dietary recall (%)**				
	1 (working day)	97.82	1.46
	2 (holiday)	98.61	2.69
	Mean (SD)	98.21 (2.68)	2.08 (2.88)
Global, mean (SD)		93.51 (6.36)	3.35 (4.33)

**Table 3:** Agreement between the Energy Balance on Cancer (BENECA) mHealth system and each gold standard assessment method.

Variable		BENECA mHealth System, mean (SD)	Gold standard method, mean (SD)	Difference of means	95% CI
**Percentage of fat**						
	Total	38.46 (8.97)	38.61 (7.59)	–0.15	–1.74 to 1.44
	Dietary record	37.44 (5.81)	38.76 (5.69)	–1.32	–2.41 to –0.23
	24-hour dietary recall	38.17 (11.50)	38.47 (11.38)	–0.29	–1.59 to 0.99
**Portions of fruits and vegetables**						
	Total	3.66 (1.91)	3.66 (1.71)	–0.01	–0.24 to 0.22
	Dietary record	3.09 (1.56)	3.03 (1.96)	0.07	–0.30 to 0.44
	24-hour dietary recall	3.89 (2.24)	4.15 (2.10)	–0.26	–0.63 to 0.11
Minutes of moderate-to-vigorous physical activity		85.51 (23.07)	86.91 (22.57)	–1.40	–3.34 to 0.55

**Table 4:** Passing-Bablok regression variables of the Energy Balance on Cancer (BENECA) mHealth system versus the 24-hour dietary recall, dietary records, and accelerometer.

Variable		Slope	95% CI	Intercept	95% CI
**Percentage of fat**						
	Total	1.22	1.03 to 1.65	–7.85	–24.58 to –1.19
	Dietary record	1.05	0.87 to 1.38	–2.84	–15.28 to 4.13
	24-hour dietary recall	1.04	0.92 to 1.20	–1.17	–8.19 to 2.94
**Portions of fruits and vegetables**						
	Total	1.11	0.98 to 1.20	–0.27	–0.58 to 0.02
	Dietary record	0.84	0.70 to 1.05	0.61	–0.01 to 1.07
	24-hour dietary recall	1.05	0.86 to 1.25	–0.19	–1.19 to 0.45
Minutes of moderate-to-vigorous physical activity		0.97	0.87 to 1.07	11.2	1.37 to 16.93

The linear regression analysis revealed coefficients of .93 (95% CI 0.88-1.34), .97 (95% CI 0.86-1.10), and .92 (95% CI 0.74-1.14), with respect to percentage of total fat, 24-hour dietary recalls, and dietary records, respectively. The coefficients for the portions of fruits and vegetables consumed were .97 (95% CI 0.95-1.22) for the total means, .94 (95% CI 0.82-1.19) for the 24-hour dietary recalls, and .93 (95% CI 0.59-0.86) for the dietary records. The model also showed a coefficient of .98 (95% CI 0.91-1.09) for the minutes of moderate-to-vigorous physical activity.

The Passing-Bablok regression analysis did not show considerable bias in percentage of fat (dietary record and 24-hour dietary recall), or portions of fruits and vegetables (Table 4). Only in terms of the percentage of total fat and minutes of moderate-to-vigorous physical activity did the analysis reveal a fixed bias without a substantial proportional bias. However, a substantial proportional bias, but not substantial fixed bias, was revealed when analyzing the percentage of total fat or moderate-to-vigorous physical activity in each assessment method (Table 4).

## Discussion

### Principal Results

The BENECA mHealth system can be used to assess the energy balance behaviors in breast cancer survivors. It is a straightforward, fast, and consistent assessment system, as shown by the results presented here. Although the BENECA mHealth system has been validated for use in breast cancer survivors, it could be used with other cancer survivors (ie, prostate or colon) because it is based on International Guidelines.

### Comparison With Prior Work

The results of this study highlighted the positive agreement between the BENECA mHealth system and daily, 24-hour dietary recalls, as well as accelerometer data (high match rate, low phantom rate). Moreover, intraclass correlation coefficient data suggested satisfactory reliability, with high coefficients for the average of the measurements. To our knowledge, since this is the only strategy that has been developed to assess energy balance in cancer survivors, it is difficult to compare our results to other investigations. Hillier et al (2012) designed SNAPA, a Web-based computer platform that can evaluate the dietary and physical activity conducts in grown-ups. However, our results were not in agreement with this study, which had a match rate of over 75% and a phantom rate below 8.6%. Our results displayed greater a match rate and a lesser phantom rate than other studies, which reported match rates between 51% and 73% [10,11,15], and phantom rates between 20% and 55% [15,43]. One possibility is that these women, who felt neglected after their medical intervention, adhered better to new technologies [44,45]. Nevertheless, the protocols and evaluation were not similar.

Similar to what has been observed with the SNAPA platform, the most commonly forgotten food in the BENECA mHealth system was “healthy” food, such as vegetables or fruits. It could be that fruits and vegetables were often forgotten because of how the dietary questionnaire in BENECA system was designed. The participants had to introduce each food separately making it easy to forget about fruit and vegetable accompaniments. In contract to our observations, there is a collective perception that people tend to record more “healthy” food and tend to forget “unhealthy” food [10]. Moreover, compared with other assessment methods that use communications and information technologies in different populations, the BENECA mHealth system shows equal or higher reliability [12-14].

### Strengths and Limitations

One of the advantages of the BENECA mHealth system is making the main gold standard methods to assess diet and physical activity readily available to patients. Moreover, the BENECA mHealth system is simple to install, compatible with commonly-used Android systems (in the future BENECA will be developed for IOS), and ease of access (Google Play Store in the future). Importantly, an internet connection is not required for its use. Despite these advantages, participants found it difficult to introduce the diet data into the BENECA system, where the grams of each individual food had to be entered. Other disadvantages included: (1) a requirement for basic mobile phone capabilities; and (2) it is only available in Spanish. Our goal is to address these disadvantages and improve future versions of the app.

Given that one of the inclusion criteria to participate in the study was to be able to use mobile apps, the average age of the participants was relatively young. Technology capacity is more common in younger breast cancer survivors, so perhaps these results may not be generalizable to older breast cancer survivors. Future studies should be conducted to clarify this issue, including a population with a higher average age.

### Clinical Implications

We believe it would be interesting to combine BENECA with some objective measurement instrument of physical activity, such as an automatic monitoring bracelet, in order to fully automate the recording of physical activity. BENECA is not only useful in clinical research to evaluate the instantaneous energy balance, but it could also be used as a tool to remotely evaluate the time change in this balance after different intervention procedures or surgical procedures. Moreover, BENECA could be used to facilitate the incorporation of physical exercise programs and healthy diet into the care system of cancer survivors. It is possible that the triangulation generated between the methods used in this trial to monitor physical activity and diet (BENECA, accelerometers, professionals) could have an educational and motivational impact on the patient. However, due to the simplicity of the app, not having to combine it with other components could produce even better results by decreasing the time required to monitor physical activity with accelerometers. Moreover, it could promote patients’ autonomy from health care professionals, lower sanitary costs, and supply motivational support through its real-time feedback system.

### Conclusions

Our preliminary results showed that the mHealth app BENECA may be a new tool to measure physical activity and intake in breast cancer survivors, in a reliable and simple way. Not only will the real-time feedback system used in BENECA enable positive changes in the lifestyles of breast cancer survivors, it can be used to motivate them to maintain these changes over time.

## Appendix

Multimedia Appendix 1 Presentation and tutorial of Energy Balance on Cancer (BENECA) mobile health system.

## Abbreviations

BENECA: Energy Balance on Cancer
BMI: body mass index
eHealth: electronic health
mHealth: mobile health
